# Machine learning-based in-hospital mortality risk prediction tool for intensive care unit patients with heart failure

**DOI:** 10.3389/fcvm.2023.1119699

**Published:** 2023-04-03

**Authors:** Zijun Chen, Tingming Li, Sheng Guo, Deli Zeng, Kai Wang

**Affiliations:** ^1^ Department of Cardiology, The Yongchuan Hospital of Chongqing Medical University, Chongqing, China; ^2^ Department of Cardiology, The Second Affiliated Hospital of Chongqing Medical University, Chongqing, China; ^3^ Department of Cardiology, The People’s Hospital of Rongchang District, Chongqing, China

**Keywords:** heart failiure, mortality, risk stratication, machine laerning, calculator, intensive care unit

## Abstract

**Objective:**

Risk stratification of patients with congestive heart failure (HF) is vital in clinical practice. The aim of this study was to construct a machine learning model to predict the in-hospital all-cause mortality for intensive care unit (ICU) patients with HF.

**Methods:**

eXtreme Gradient Boosting algorithm (XGBoost) was used to construct a new prediction model (*XGBoost model*) from the Medical Information Mart for Intensive Care IV database (MIMIC-IV) (training set). The eICU Collaborative Research Database dataset (eICU-CRD) was used for the external validation (test set). The XGBoost model performance was compared with a logistic regression model and an existing model (Get with the guideline-Heart Failure model) for mortality in the test set. Area under the receiver operating characteristic cure and Brier score were employed to evaluate the discrimination and the calibration of the three models. The SHapley Additive exPlanations (SHAP) value was applied to explain XGBoost model and calculate the importance of its features.

**Results:**

The total of 11,156 and 9,837 patients with congestive HF from the training set and test set, respectively, were included in the study. In-hospital all-cause mortality occurred in 13.3% (1,484/11,156) and 13.4% (1,319/9,837) of patients, respectively. In the training set, of 17 features with the highest predictive value were selected into the models with LASSO regression. Acute Physiology Score III (APS III), age and Sequential Organ Failure Assessment (SOFA) were strongest predictors in SHAP. In the external validation, the XGBoost model performance was superior to that of conventional risk predictive methods, with an area under the curve of 0.771 (95% confidence interval, 0.757–0.784) and a Brier score of 0.100. In the evaluation of clinical effectiveness, the machine learning model brought a positive net benefit in the threshold probability of 0%–90%, prompting evident competitiveness compare to the other two models. This model has been translated into an online calculator which is accessible freely to the public (https://nkuwangkai-app-for-mortality-prediction-app-a8mhkf.streamlit.app).

**Conclusion:**

This study developed a valuable machine learning risk stratification tool to accurately assess and stratify the risk of in-hospital all-cause mortality in ICU patients with congestive HF. This model was translated into a web-based calculator which access freely.

## Introduction

Congestive heart failure (HF) is a complex clinical syndrome caused by structural and/or functional disorders. These disorders lead to high mortality rate in HF patients. Despite significant advances in its treatment in recent years, HF mortality has not yet reached a decreasing inflection point (1). In the United States, approximately 10% of hospitalized patients with HF require admission to the intensive care unit (ICU) for a higher level of intensive care (2, 3), which imposes a heavy economic and social burden. The risk stratification of the ICU patients has become an important strategy in management of HF. Studies have also demonstrated that accurate event risk assessment and active intervention can help improve cardiac disease outcomes (4). Therefore, effective stratification tools are needed to achieve this goal. The Get with the guideline-Heart Failure (*GWTG-HF model*) risk score uses common clinical features (race and systolic blood pressure) to predict in-patient mortality. However, it was developed more than a decade ago and has unsatisfactory applicability in current clinical practice (5, 6). Additionally, with the increasing availability of clinical data, traditional prediction models based on logistic regression analysis may not be able to capture nonlinear relationships from high-dimensional data.

Machine learning algorithms, which provide researchers with powerful tools, have been applied in several medical fields, ranging from disease diagnosis, outcome prediction, and efficacy prediction to medical image interpretation (7–9). It has also been used to develop new HF risk prediction models. These models show a better prediction performance (7–9). However, most of these models were verified only in the same cohort, and independent external verification was seldom performed. Moreover, the degree of model calibration has not been reported (8, 10). Based on the above, in this study, a machine learning algorithm was introduced to construct an in-patient mortality risk prediction model for ICU HF patients, and an independently validation was performed in the test dataset.

## Methods

### Study population

**Figure 1:**
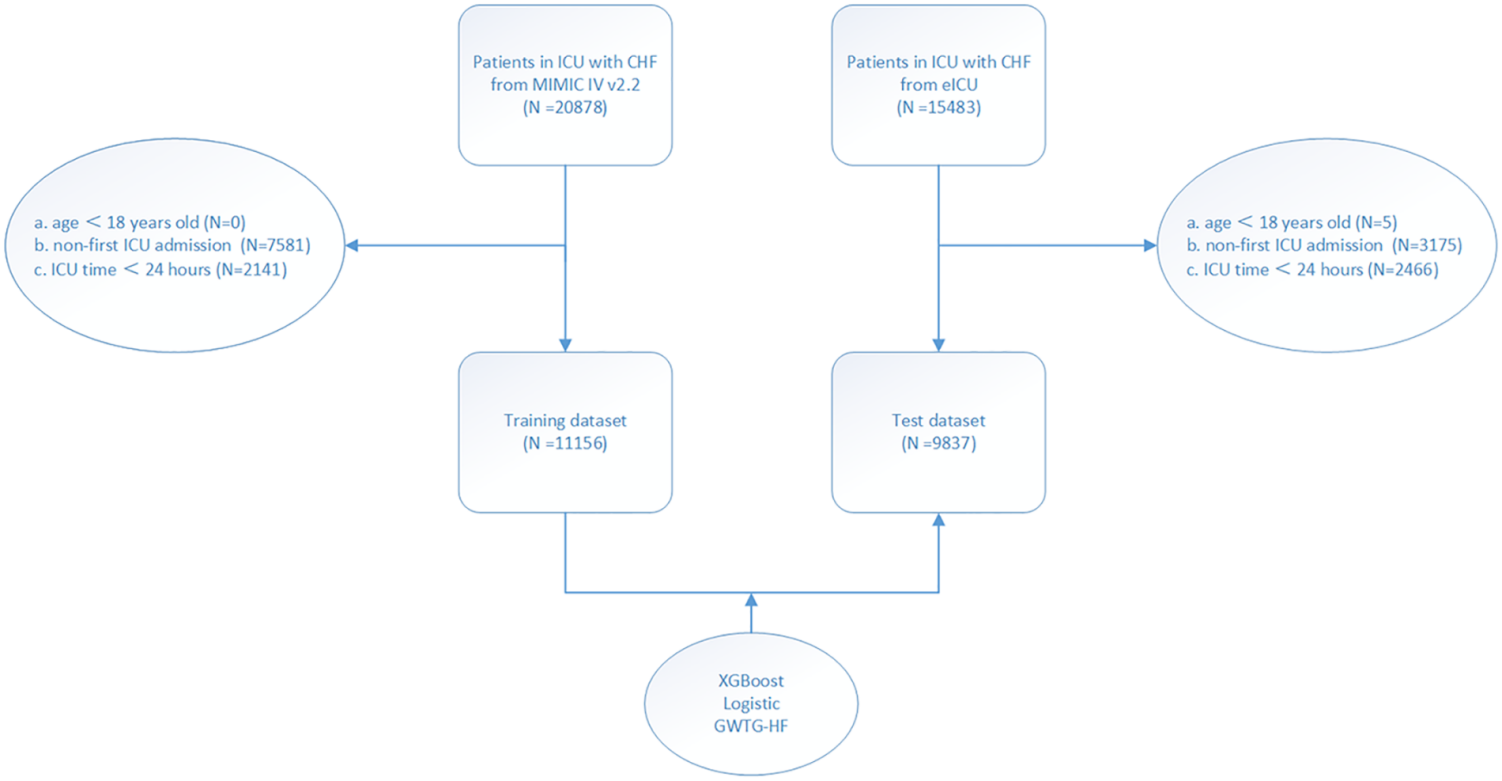
Study flow chart. According to the inclusion and exclusion criteria of the study, the population of the training set and the test set were obtained.

The study population was enrolled from two different databases: The Medical Information Mart for Intensive Care IV database (MIMIC-IV) and the eICU Collaborative Research Database dataset (eICU-CRD). MIMIC-IV (version 2.2) is a single-center database that contains data from over 190,000 ICU patients between 2008 and 2019, including demographic records, hourly vital signs from bedside monitors, laboratory tests, international classification of diseases (ICD-9 and ICD-10) code diagnostics, and other clinical features (11). The eICU-CRD is a multi-center intensive care database that contains data of 335 ICU patients in the United States from 2014 to 2015 (12). Inclusion criteria: All patients with congestive heart failure. The data of patients with congestive heart failure were obtained from the two databases by ICD-9 and ICD-10 code (Supplementary Material). Exclusion criteria: (a) patients less than 18 years of age; (b) patients not admitted to ICU for the first time; (c) patients with ICU stay less than 24 h. Ultimately, as shown in the flow chart (Figure 1), 11,156 and 9,837 patients were enrolled in the study from the MIMIC-IV and eICU-CRD, respectively. This study was a secondary analysis based on the data described above, and all patient’ information was anonymized; therefore, the requirement of informed patient consent was waived.

### Data acquisition and outcome definition

According to previous researches (13, 14), this study documented demographic characteristics (age, sex, weight and height), severity score [Sequential Organ Failure Assessment (SOFA) and Acute Physiology Score III (APS III)], comorbidities [diabetes, hypertension, severe liver disease, chronic obstructive pulmonary disease (COPD), and myocardial infarction], medication information [dopamine, norepinephrine, dobutamine, epinephrine, phenylephrine, vasopressin, milrinone, furosemide, beta blocker and angiotensin converting enzyme I (AECI)/Angiotensin II receptor blocker (ARB)], mechanical ventilation, continuous renal replacement therapy (CRRT), vital signs at admission (heart rate, respiratory rate, SpO_2_, systolic blood pressure and mean blood pressure), and laboratory tests [white blood cell count (WBC), basophils, eosinophils, lymphocytes, monocytes, neutrophils, red blood cells count (RBC), hematocrit, hemoglobin, mean corpuscular volume (MCV), mean corpuscular hemoglobin content (MCHC), mean hemoglobin concentration (MHC), red blood cell distribution width (RDW), platelets, albumin, aspartate aminotransferase (AST) alanine aminotransferase (ALT), alkaline phosphatase (ALP), total bilirubin, urea nitrogen (BUN), creatinine, glucose, sodium, calcium, chloride, potassium, anion gap, bicarbonate, international normalized ratio (INR), thromboplastin time (PTT), prothrombin time (PT)] at admission. The same features in eICU-CRD were extracted to achieve external validation of those models. The primary endpoint was defined as all-caused in-hospital mortality based on survival at discharge. Data extraction process was performed using the PostgreSQL programming language.

### Model construction and validation

The MIMIC-IV and eICU-CRD datasets were used as training set and test set, respectively. In the training set, the Least Absolute Shrinkage and Selection Operator (LASSO) regression was used to select the features from items (15). The eXtreme Gradient Boosting (XGBoost) is an optimal implementation of gradient boosting which is based on the ensemble of weak learners with high bias and low variance (15). Taking the primary endpoint and the screened features as the prediction outcome and the prediction features, respectively, XGBoost was applied to obtain the optimal model super parameters (such as the number of trees, the maximum depth of the trees, the learning rate, and the like) through 10-fold cross-validation and Bayesian optimization. This machine learning model designated as *XGBoost model*. Logistic regression was applied to obtain a generalized linear regression model (*Logistic model*). The *GWTG-HF model* was obtained according to the previous study (6).

Machine learning models are considered black boxes due to difficulty in explaining how an algorithm provides accurate predictions for a specific population. Therefore, we introduced the SHapley Additive exPlanations (SHAP) value to explain the *XGBoost model* and calculate the importance of its features. SHAP is a unified framework for explaining machine learning algorithm prediction proposed by Lundberg and Lee as a new method for explaining various black box models, with verified interpretable properties (16).

Subsequently, the *XGBoost model*, *Logistic model* and *GWTG-HF model* were independently validated and compared with each other in the test set. Model discrimination was assessed using the area under the receiver operating characteristic curve (AUC). The calibration evaluation of the model was assessed using the calibration map and the Brier score. Clinical effectiveness evaluation of models was assessed by using decision curve analysis.

### Statistical analysis

Continuous variables are expressed as mean and standard deviation, and the *t*-test was used for comparisons between the groups. Categorical variables are expressed as absolute values and proportions, and the chi-squared test was used for comparison between the groups. In this study, it was assumed that the missing clinical features occurred randomly, and multiple interpolations were performed using the random forest method in the multivariate imputation by chained equation (MICE) function of the MICE package, assuming missing clinical features. *P* value <0.05 was considered as statistically significant. All statistical analyses were performed using R 4.1.3 software (The R Foundation for Statistical Computing, Vienna, Austria).

## Results

### Population baseline characteristics

A total of 11,156 and 9,837 HF patients from the MIMIC-IV (training set) and eICU-CRD (test set), were included in the study respectively (Table 1). Among them, the median age was 73 and 71 years, and 55.9% and 53.0% were men, respectively. The proportions of patients with diabetes, hypertension, severe liver disease, COPD and myocardial infarction were 40.3%, 77.2%, 2.9%, 20.4%, 32.9%, 40.8%, 66.4%, 1.3%, 20.4% and 17.3%, respectively. The proportions of patients requiring mechanical ventilation were 39.3% and 46.4%, respectively. Endpoint events occurred in 13.3% (1,484/11,156) and 13.4% (1,319/9,837) of patients, respectively.

**Table 1:** Clinical data of the study population.

	Medical information mart for intensive care IV v2.2 database (training set)	eICU Collaborative research database (test set)	*P* value
Total, *n*	11,156	9,837	
Age [mean (SD)]	72.97 (13.62)	70.66 (13.64)	<0.001
Male, *n* (%)	6,238 (55.9)	5,210 (53.0)	<0.001
BMI [mean (SD)]	28.92 (6.62)	29.65 (7.27)	<0.001
Diabetes, *n* (%)	4,493 (40.3)	4,016 (40.8)	0.425
Hypertension, *n* (%)	8,610 (77.2)	6,533 (66.4)	<0.001
Severe_liver_disease, *n* (%)	320 (2.9)	129 (1.3)	<0.001
COPD, *n* (%)	2,274 (20.4)	2,009 (20.4)	0.958
Myocardial_infarct, *n* (%)	3,667 (32.9)	1,700 (17.3)	<0.001
SOFA [mean (SD)]	5.83 (3.62)	5.51 (3.06)	<0.001
APS III [mean (SD)]	51.90 (22.52)	47.44 (22.70)	<0.001
Heart rate [mean (SD)]	88.24 (20.31)	91.26 (22.86)	<0.001
Respiratory rate [mean (SD)]	19.84 (6.19)	22.03 (6.60)	<0.001
SpO_2_ [mean (SD)]	96.58 (4.29)	95.17 (6.65)	<0.001
MBP [mean (SD)]	80.47 (18.78)	85.59 (21.91)	<0.001
WBC [mean (SD)]	12.37 (8.67)	11.52 (6.46)	<0.001
Hematocrit [mean (SD)]	33.00 (7.14)	35.74 (7.26)	<0.001
Hemoglobin [mean (SD)]	10.71 (2.38)	11.57 (2.44)	<0.001
Platelets [mean (SD)]	215.44 (106.62)	226.82 (105.98)	<0.001
RBC [mean (SD)]	3.62 (0.82)	3.96 (0.83)	<0.001
MCH [mean (SD)]	29.77 (2.77)	29.34 (2.92)	<0.001
MCV [mean (SD)]	91.77 (7.34)	90.69 (7.75)	<0.001
MCHC [mean (SD)]	32.46 (1.71)	32.34 (1.57)	<0.001
RDW [mean (SD)]	15.42 (2.32)	16.06 (2.49)	<0.001
Basophils [mean (SD)]	0.03 (0.04)	0.07 (0.08)	<0.001
Eosinophils [mean (SD)]	0.11 (0.24)	0.19 (0.30)	<0.001
Lymphocytes [mean (SD)]	1.57 (4.45)	1.52 (2.49)	0.323
Monocytes [mean (SD)]	0.65 (0.69)	0.83 (0.64)	<0.001
Neutrophils [mean (SD)]	10.11 (6.21)	8.81 (5.32)	<0.001
Albumin [mean (SD)]	3.27 (0.61)	3.18 (0.61)	<0.001
ALT [mean (SD)]	83.68 (333.33)	71.36 (327.86)	0.007
AST [mean (SD)]	129.44 (596.34)	91.44 (444.39)	<0.001
ALP [mean (SD)]	109.72 (104.02)	110.85 (86.86)	0.399
Total bilirubin [mean (SD)]	1.06 (1.92)	0.93 (1.09)	<0.001
BUN [mean (SD)]	33.95 (24.96)	35.06 (23.96)	0.001
Creatinine [mean (SD)]	1.74 (1.68)	1.96 (1.93)	<0.001
Glucose [mean (SD)]	154.98 (89.08)	163.11 (93.86)	<0.001
Sodium [mean (SD)]	137.96 (5.41)	137.05 (5.47)	<0.001
Calcium [mean (SD)]	8.50 (1.08)	8.75 (0.79)	<0.001
Chloride [mean (SD)]	102.13 (6.97)	100.65 (6.64)	<0.001
Potassium [mean (SD)]	4.41 (0.86)	4.34 (0.81)	<0.001
Aniongap [mean (SD)]	15.74 (4.72)	11.75 (5.00)	<0.001
Bicarbonate [mean (SD)]	23.57 (5.22)	25.82 (5.85)	<0.001
INR [mean (SD)]	1.66 (1.26)	1.68 (1.17)	0.185
PT [mean (SD)]	18.02 (12.72)	18.79 (11.96)	<0.001
PTT [mean (SD)]	40.47 (26.22)	36.92 (18.51)	<0.001
CRRT [mean (SD)]	0.06 (0.23)	0.07 (0.25)	0.001
Dopamine, *n* (%)	507 (4.5)	294 (3.0)	<0.001
Norepinephrine, *n* (%)	2,436 (21.8)	1,027 (10.4)	<0.001
Dobutamine, *n* (%)	317 (2.8)	327 (3.3)	0.047
Phenylephrine, *n* (%)	2,102 (18.8)	252 (2.6)	<0.001
Epinephrine, *n* (%)	956 (8.6)	111 (1.1)	<0.001
Vasopressin, *n* (%)	622 (5.6)	184 (1.9)	<0.001
Milrinone, *n* (%)	382 (3.4)	211 (2.1)	<0.001
Blocker, *n* (%)	5,953 (53.4)	1,788 (18.2)	<0.001
ACEI/ARB, *n* (%)	515 (4.6)	453 (4.6)	0.007
Furosemide, *n* (%)	5,517 (49.5)	2,507 (25.5)	<0.001
Ventilation, *n* (%)	4,379 (39.3)	4,567 (46.4)	<0.001
In-hospital mortality, *n* (%)	1,484 (13.3)	1,319 (13.4)	0.837

BMI, body mass index; SOFA, sequential organ failure assessment; APS III, acute physiology score III scores; COPD, chronic obstructive pulmonary disease; AECI/ARB, angiotensin converting enzyme I/Angiotensin II receptor blocker; CRRT, continuous renal replacement therapy; WBC, white blood cell count; RBC, red blood cells count; MCV, mean corpuscular volume; MCHC, mean corpuscular hemoglobin content; MHC, mean hemoglobin concentration; RDW, red blood cell distribution width; AST, aspartate aminotransferase; ALT, alanine aminotransferase; ALP, alkaline phosphatase; BUN, urea nitrogen; INR, international normalized ratio; PTT, thromboplastin time; PT, prothrombin time.

### Feature selection

**Figure 2:**
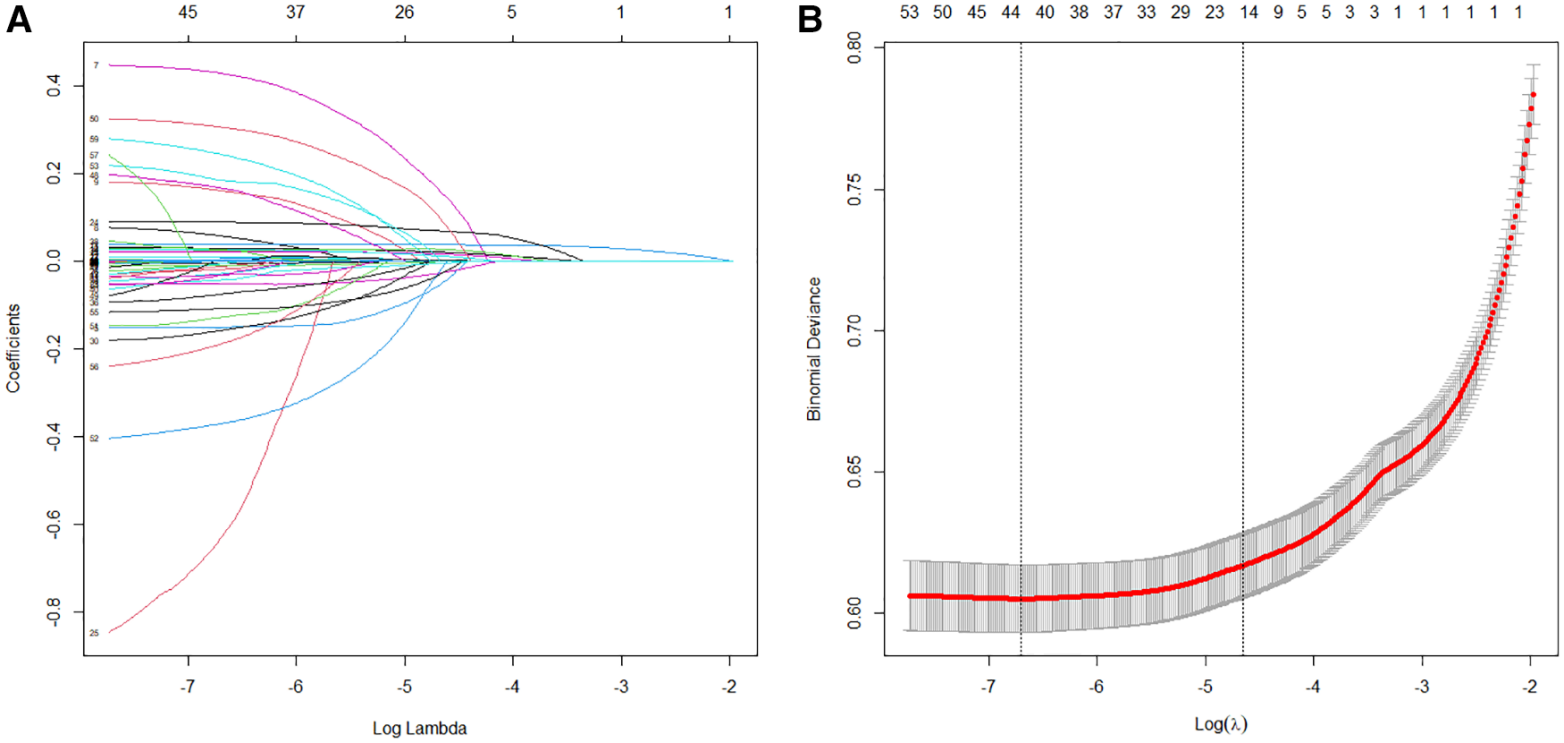
Features selection process. Automated feature selection for 57 clinical factors was performed using Least Absolute Shrinkage and Selection Operator, which minimized the loss function binomial deviance, shrank coefficients, and produced some coefficients that are zero, allowing efficient feature selection (**A**). The algorithm outputted 17 filtered features with non-zero coefficients that were included in model generation subsequently (**B**).

In the training set, the LASSO regression was used for the automatic features selection (Figure 2). LASSO regression minimizes the loss function (binomial deviance) by changing the regularization coefficient lambda (*λ*) to generate zero coefficients. Finally, 17 features with the highest predictive value were introduced into the models.

### Model establishment construction

**Figure 3:**
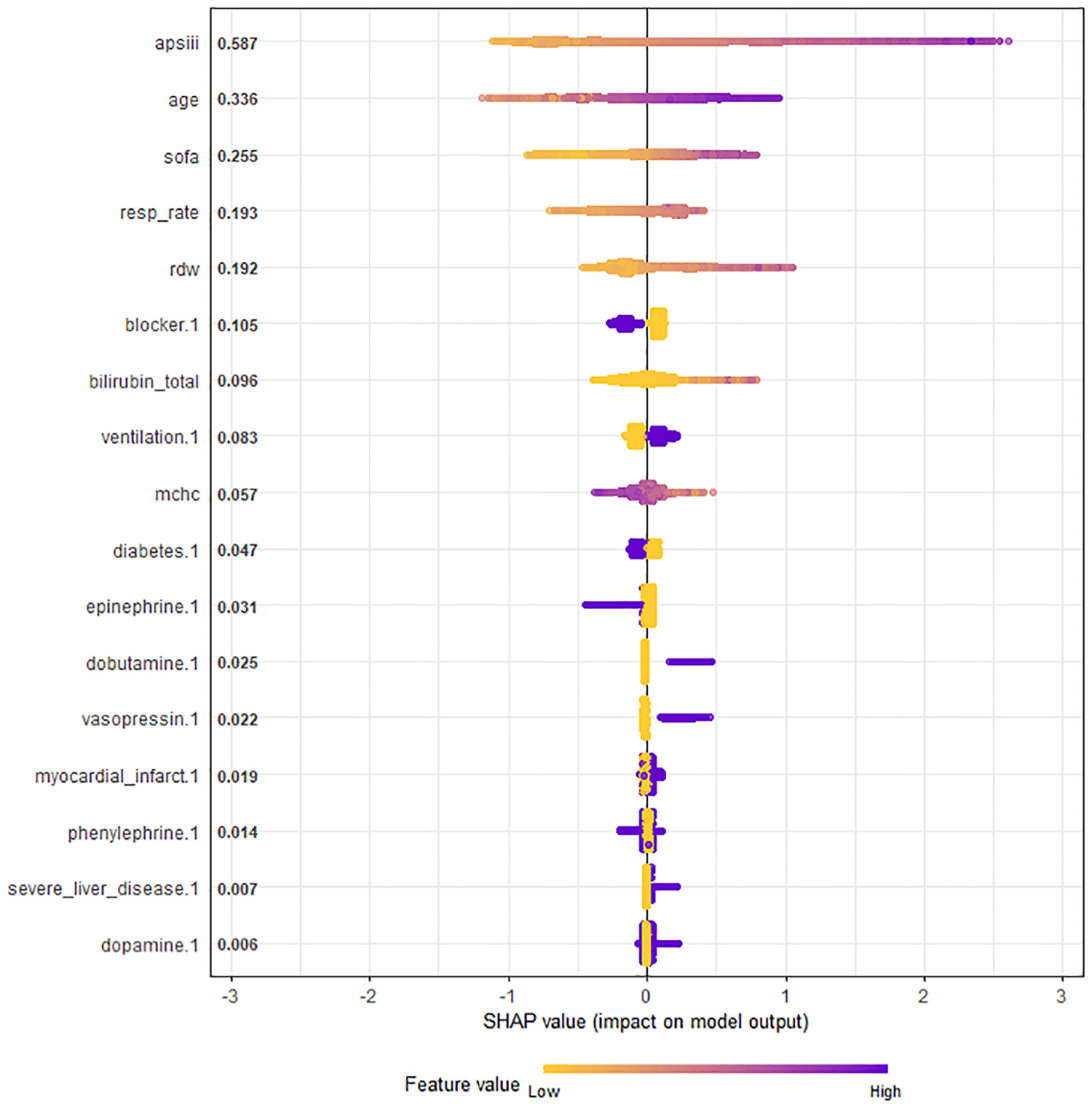
Chart of feature importance ranking in all ICU patients with congestive heart failure. The importance ranking of the 17 risk factors with stability and interpretation using the optimal model in the training set. Each point in the graph represents the SHAP value for each sample; a color month closer to purple indicates a larger value, while that closer to yellow indicates a smaller value. The more scattered the points in the graph, the greater the influence of the variable on the model. APS III, age and SOFA were strongest predictors. SHAP, SHapley additive exPlanations; SOFA, sequential organ failure assessment; APS III, acute physiology score III scores; MCHC, mean corpuscular hemoglobin content; RDW, red blood cell distribution width.

The selected 17 features were input into a machine learning algorithm to establish the *XGBoost model* in the training set. The same procedure was performed using the traditional linear regression method to establish the *Logistic model*. The super-parameters of the *XGBoost model* were obtained: number of trees (nrounds) = 95; maximum depth of the tree (max depth) = 3; learning rate (eta) = 0.1527683; sample resampling ratio (subsample) = 0.6293208; minimum loss split (gamma) = 0.1934144; minimum sample weight required on child nodes (min childweight) = 10; characteristic random sampling ratio (colsample bytree) = 0.6447264; and the single maximum increment allowed in the weight estimation of the tree (max delta step) = 10. The importance of each prediction feature of the *XGBoost model* was obtained using the SHAP algorithm, and the importance graph listed the features in descending order (Figure 3).

The SHAP values represented the contribution of each feature to the final prediction and are useful for elucidating and interpreting model predictions for a single patient. The combined effect of all factors provided the final SHAP value that corresponded to the predicted score. APS III, age and SOFA were the three strongest predictors (Figure 3).

### Model validation

**Figure 4:**
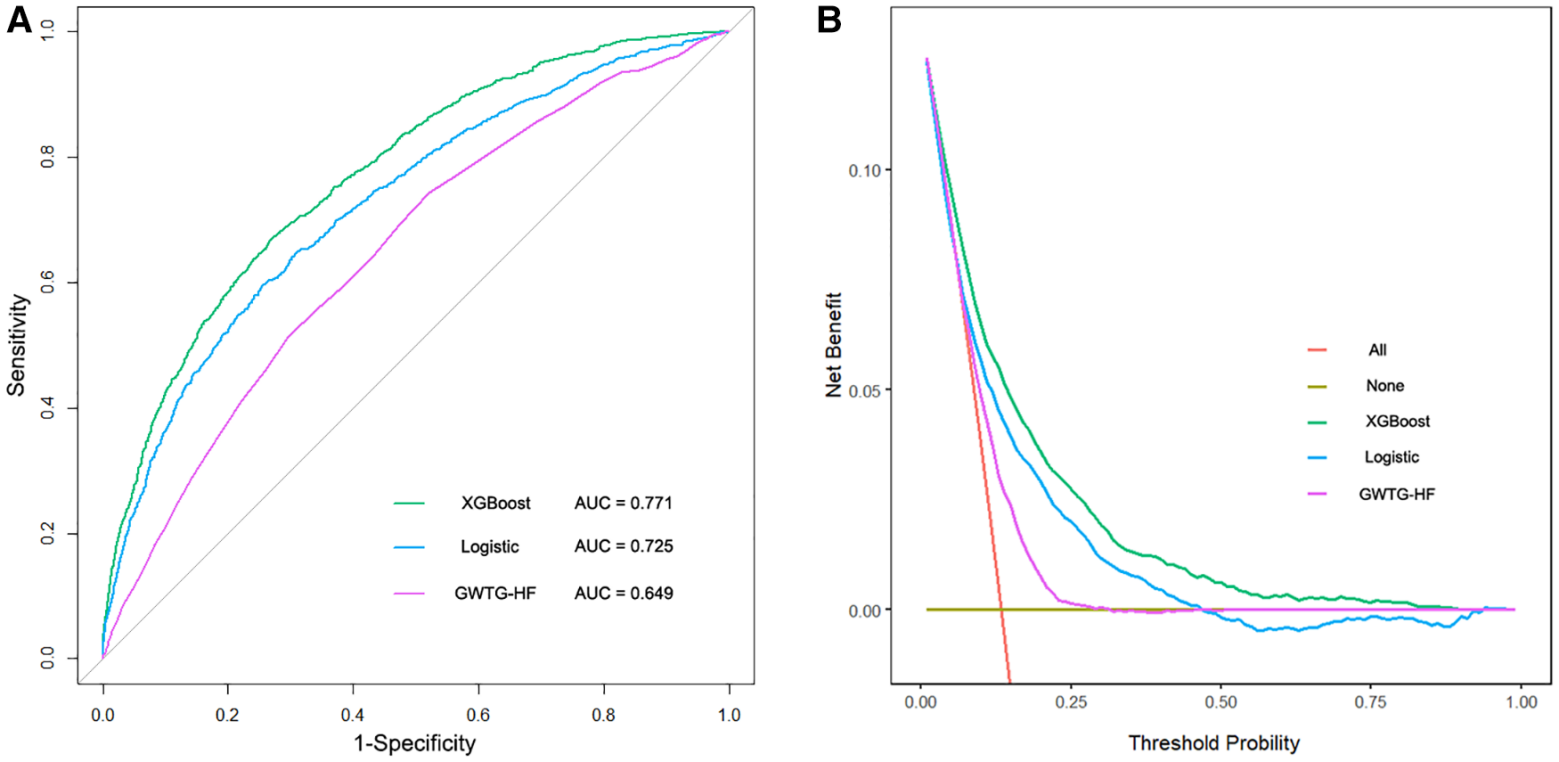
The receiver operating characteristic curves and the decision curve analysis of all models in test dataset. (**A**). The brown transverse line = net benefit when all patients are considered to not have the outcome (in-hospital all-cause mortality); red oblique line = net benefit when all patients were considered to have the outcome; the decision curve analysis of all models showed that the proportion of the benefit for the population was the highest when the risk assessment of the *XGBoost model* was used for treatment, while the treatment threshold probability was from 0% to 90% (**B**).

**Figure 5:**
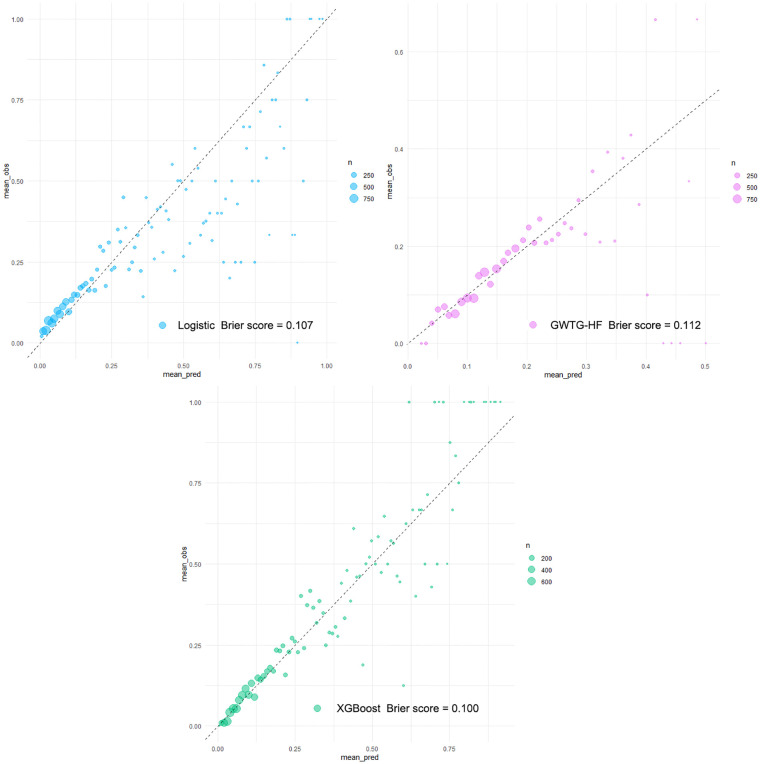
Calibration plots of all models in testing dataset. Calibration plots of predicted probabilities (*X*-axis) and actual proportions (*Y*-axis) for different prediction models. With the calibration slope closest to 1.0 (an ideal model), the eXtreme Gradient Boosting (*XGBoost model*) from the machine learning algorithm obtained a fairly satisfactory calibration, while the other models calibrated poorly.

The *XGBoost model*, *Logistic model*, and *GWTG-HF model* were independently validated in the test set. The *XGBoost model* performed best in the discrimination evaluation (Figure 4A), with an AUC of 0.771 [95% confidence interval (CI): 0.757–0.784] compared to the *Logistic model* (AUC 0.725, 95% CI: 0.710–0.740, *P *< 0.001) and the *GWTG-HF model* (AUC 0.649, 95% CI: 0.633–0.665, *P* < 0.001). The calibration degree evaluation was performed to demonstrate the consistency between the predicted probability of the model and the actual probability. The results showed that the three models had a good degree of calibration (Figure 5). The *XGBoost model* achived better Brier scores than the *Logistic model* and the *GWTG-HF model* (Brier score: 0.100, 0.107 and 0.112, respectively), which indicated better model calibration. In addition, in the evaluation of clinical effectiveness, the analysis of the decision curve showed that the net benefit level using the *XGBoost model* was higher than “zero risk of mortality” and “all mortality”, and superior to the other two models in the threshold probability of 0%–90% (Figure 4B).

### Model deployment

**Figure 6:**
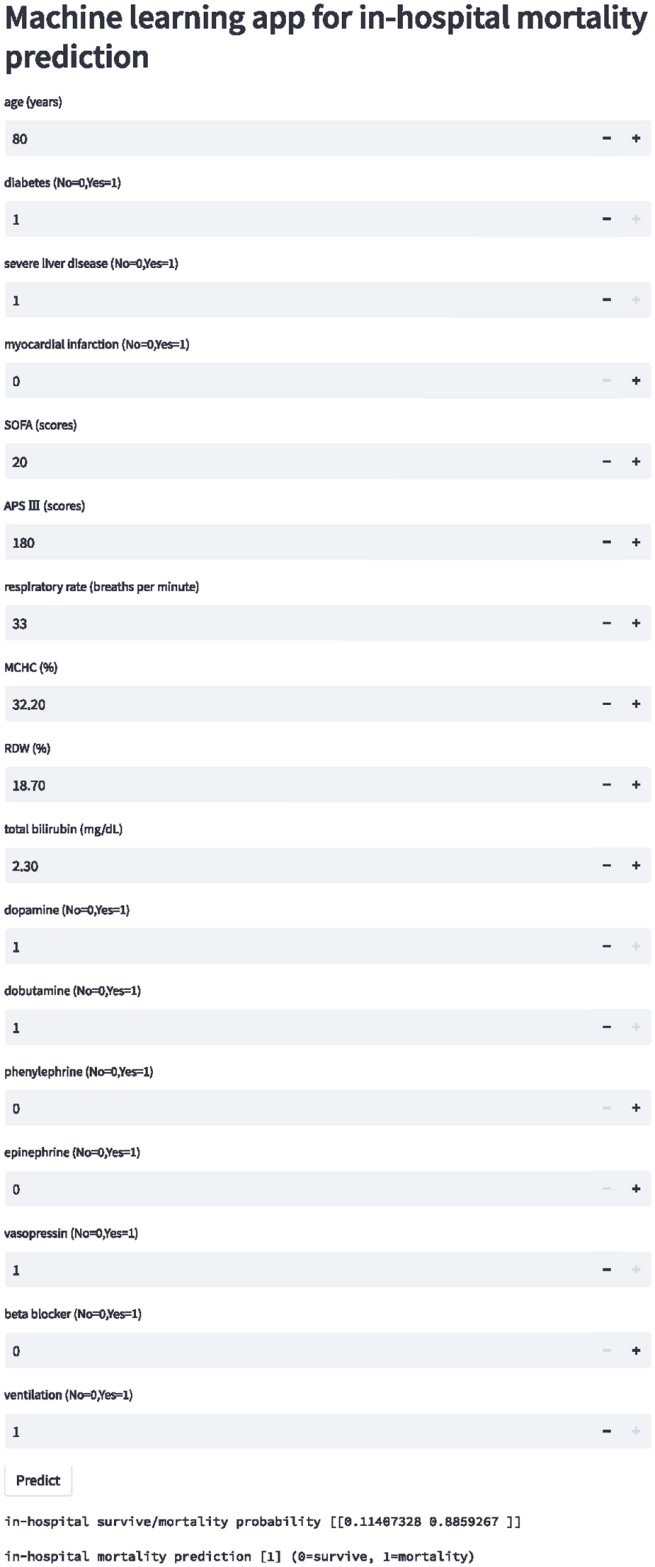
Schematic diagram of online website calculator for the mortality prediction in all ICU patients with congestive heart failure. Input the patient information and click the “predict” button to get the patient's in-hospital mortality risk assessment results.

An internet-based version of the *XGBoost model* has been accessible for all physicians (https://nkuwangkai-app-for-mortality-prediction-app-a8mhkf.streamlit.app). This web-based tool will automatically predict the outcome for ICU patients with HF when the values of the 17 features required for this model are entered as shown in Figure 6. Moreover, the online tool provides users with the explanation of the prediction of the model. It can predict the outcome of patients with missing values.

## Discussion

In this study, we established and validated an interpretable machine learning-based risk stratification tool for in-hospital all-cause mortality in ICU HF patients. Compared with traditional risk prediction methods, machine learning technology captures both the linear and the nonlinear relationships between risk prediction factors and mortality endpoints from high-dimensional datasets. Our model achieved the most satisfactory risk stratification and calibration.

Data from 341 hospitals in the United States showed the median ICU admission rate for hospitalized HF patients was 10% (interquartile range, 6%–16%) (3), and the in-hospital mortality rate of HF patients admitted to the ICU was significantly higher than that of patients in general wards. The all-cause in-hospital mortality rates were 17.3% and 6.5% for ICU-admitted HF patients and all HF patients, respectively, in the RO-AHFS study (17), and 17.8% and 4.5% in the ALARM-HF study (18). The in-hospital mortality rates in our study were 13.3% and 13.4%, respectively, similar to those in the above studies. Although the high in-hospital mortality in ICU HF patients was primarily due to the underlying disease severity, accurate prognostic evaluation is the basis of clinical decision-making in ICU patients with HF. Therefore, this study has potentially important clinical implications.

A major challenge in the management of HF is the identification of mortality risk. Those previously prediction models (*GWTG-HF model* and *Logistic model*) were mostly based on traditional generalized linear regression, such as logistic regression, which is highly explanatory. However, faced with a large data volume and high data latitude, they may be useless, as the clinical processes are often unpredictable and the clinical factors of HF are highly variable, which makes it difficult for physicians to obtain accurate predictions using single-factor analysis. The application of machine learning integrated provides more powerful support for clinical decision-making (19–21). Similar to these studies, our study obtained a novel *XGBoost model* by applying a more flexible integrated machine learning algorithm. This model was derived from a comprehensive consideration of clinical features, comorbidities, and medication. Distinct from other machine learning models (7–9, 13, 22, 23), our study established an independent external test set and reported the calibration degree of the models in detail. Independent validation confirmed that our *XGBoost model* significantly improved prediction performance compared with the *Logistic model* and the *GWTG-HF model* in all-cause mortality risk for ICU patients with HF. It is worth mentioning that population characteristics between the training set and the test set were significantly different, but our *XGBoost model* overcame this difference and achieved good risk stratification (Table 1, Figures 4, 5). This excellent risk stratification performance may indicate that our *XGBoost model* can be generalized to other HF patients, not just the test set. This improvement also confirmed the nonlinear correlation between the clinical features of patients with HF and the risk of in-hospital mortality from another perspective. The good prediction alerts doctors and patients to the disease’s the severity to prepare for higher-level life support, such as mechanical circulatory support and heart transplantation or hospice care. This finding requires confirmation in future studies.

Another important challenge is capacity building for the primary management of HF. With the formation of a hierarchical diagnosis and treatment system, most primary hospitals have set up ICU wards to admit critically ill patients. Previous studies attempted to incorporate more biochemical indicators or cardiac magnetic resonance imaging parameter into models to obtain superior prediction performance (24). However, imaging parameters limited their generalized application to a certain extent. In contrast, in our study, 17 variables with the most predictive value were screened into the model by LASSO, which were all easily accessible, making it possible for primary hospitals to accurately assess patients’ risk through feature acquisition.

The third obvious challenge is to correctly explain the machine learning prediction model and visually present the prediction results to clinicians. We applied the SHAP value to the *XGBoost model* for optimize prediction and interpretability. SHAP can perform both local and global interpretability, and has a solid theoretical basis compare to other methods (25). SHAP’s evaluation includes the importance of the output of all the combinations of elements, and provides a consistent and locally accurate attribute value for each element in the prediction model. This visual interpretation was applied to the black box tree integration model *XGBoost*. Furthermore, we developed a website calculator to help physicians intuitively understand both the key features and the decision-making process. These means may promote and strengthen individualized treatment strategies.

The fourth challenge is how to translate machine learning model into clinical practical solution. Few studies develop a website or application to facilitate the accession to the tool (26). We built a new website calculator to public as shown in Figure 6 (https://nkuwangkai-app-for-mortality-prediction-app-a8mhkf.streamlit.app). This calculator concisely displays the risk of in-hospital all-cause mortality in ICU patients with HF.

Inevitably, this study had several limitations. First, there were some missing values in the dataset, but multiple imputation methods were used to fill in the missing values, which may make the them closer to the true values. Second, the information collected was structured or tabular. Further studies are needed to mine and integrate the unstructured data such as medical records and imaging biomarkers to improve the prediction. Third, since out data are derived from ICU patients, this model is primarily applicable for ICU patients accompanied with HF. The clinical characteristics of ICU patients with HF might be different from those admitted to the cardiovascular care unit, so evidence is needed for the application of this model in cardiovascular care unit patients with HF. Fourth, it is difficult to tell whether or not the heart failure is the primary cause of ICU admission in MIMIC-IV. Although we validated our model in an independent external data, further research is needed to validate this model in patients with different heart failure etiologies.

## Conclusion

In this study, machine learning techniques were used to build a new risk stratification model to stratified the risk of in-hospital all-cause mortality in ICU patients with HF. This model was translated into a web-based calculator which can accessed freely. We believe that this stratification model and calculator provide a clear explanation for individualized risk prediction and serve as a simple rapid estimation tool.

## Data Availability

The original contributions presented in the study are included in the article/**Supplementary Material**, further inquiries can be directed to the corresponding author.
