# Supplementary material for: Machine learning-based in-hospital mortality risk prediction tool for intensive care unit patients with heart failure
Source: Front Cardiovasc Med. 2023 Apr 3;10:1119699. doi: 10.3389/fcvm.2023.1119699 (PMC10106627; doi:10.3389/fcvm.2023.1119699)
Supplement: Supplementary file 1 [file Datasheet1.docx]

Supplementary Material

Machine learning-based in-hospital mortality risk prediction tool for intensive care unit patients with heart failure.

**Zijun Chen, Tingming Li, Sheng Guo, Deli Zeng, Kai Wang^*^**

*** Correspondence:**Kai Wang [nkuwangkai@163.com](mailto:nkuwangkai@163.com)

ICD-9 and ICD-10 code were used to screen of patients with heart failure

| ICD-9 | | ICD-10 |
| --- | --- | --- |
| 428 | 4284 | I43 |
| 4280 | 42840 | I50 |
| 4281 | 42841 | I099 |
| 4282 | 42842 | I110 |
| 42821 | 42843 | I130 |
| 42822 | 4289 | I132 |
| 42823 | 39891 | I255 |
| 4283 | 40201 | I420 |
| 42830 | 40211 | I425 |
| 42831 | 40291 | I426 |
| 42832 | 40401 | I427 |
| 4254 | 40403 | I428 |
| 4255 | 40411 | I429 |
| 4257 | 40413 | P290 |
| 4258 | 40491 |  |
| 4259 | 40493 |  |

Note: 4280, I502-I5043 were used in eICU dataset
